# First person – Daniel Sobrido-Cameán

**DOI:** 10.1242/dmm.039214

**Published:** 2019-02-20

## Abstract

First Person is a series of interviews with the first authors of a selection of papers published in Disease Models & Mechanisms (DMM), helping early-career researchers promote themselves alongside their papers. Daniel Sobrido-Cameán is first author on ‘Serotonin inhibits axonal regeneration of identifiable descending neurons after a complete spinal cord injury in lampreys’, published in DMM. Daniel is a PhD student in the lab of María Celina Rodicio and Antón Barreiro-Iglesias at Universidade de Santiago de Compostela, Santiago de Compostela, Spain, investigating the mechanisms involved in the development and regeneration of the nervous system.


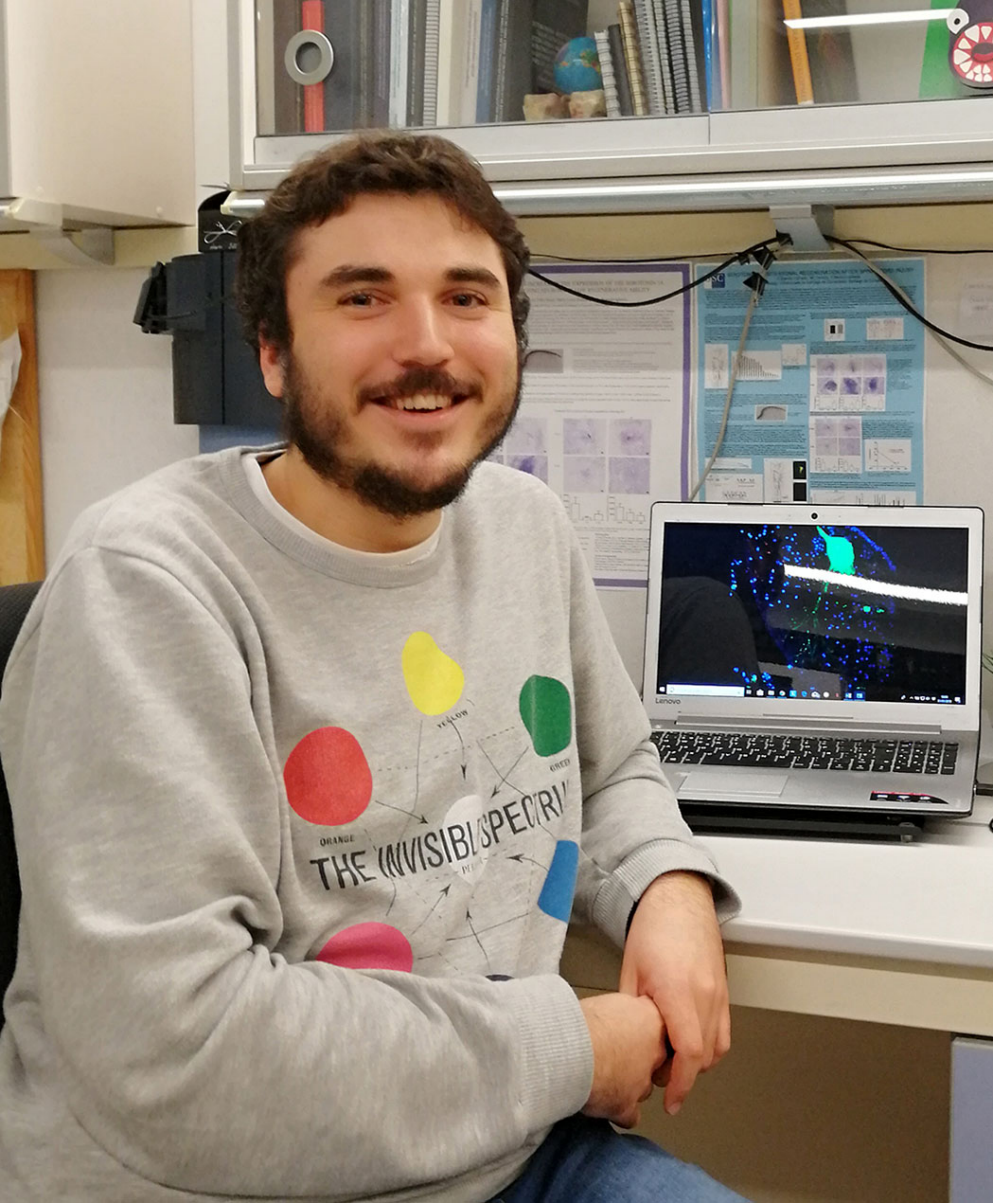


**Daniel Sobrido-Cameán**

**How would you explain the main findings of your paper to non-scientific family and friends?**

Humans suffer an irreversible loss of sensory and locomotor function after a spinal cord injury. In contrast to humans, lampreys recover the ability to swim following a complete spinal cord injury. The spinal cord is formed, among other things, by descending axons coming from the brain. Axons are the ‘wires’ used by neurons to send orders to the muscles to perform movements. Spinal cord injuries damage these axons; therefore, the ability to control muscles below the site of injury is lost. Axons of lampreys are able to regenerate after injury, which allows them to recover swimming capacity after a spinal cord injury. We use lampreys to find molecules that control axon regeneration. Serotonin is a molecule used by neurons as a signal to ‘talk to each other’. Neurons have special ‘antennas’ to receive these signals. These ‘antennas’ are known as receptors. Neurons express serotonin receptors to respond to serotonin. In our study, we show that serotonin, acting through one of its receptors, inhibits axon regeneration after spinal cord injury in lampreys. Moreover, we demonstrated that, if we inhibit the activation of these receptors using drugs, we can improve axon regeneration. I believe that my research will provide a new way to design potential treatments for spinal cord injury.

**What are the potential implications of these results for your field of research?**

In this work we demonstrate the role of endogenous serotonin in the regeneration of lamprey axons following spinal cord injury. This work provides a possible new way to promote regeneration after spinal cord injury in non-regenerating animal models. Moreover, there are treatments that modulate the serotonergic system that are used in humans, for example to treat depressive disorders, and their effects in neurological recovery after spinal cord injury are unknown. This work opens the door to study other possible uses of these drugs.

**What are the main advantages and drawbacks of the model system you have used as it relates to the disease you are investigating?**

Lampreys recover locomotion spontaneously after a complete spinal cord injury, but, in spite of the amazing regenerative capacity of their central nervous system, not all neurons show the same abilities for axon regeneration. Among the brain descending neurons of lampreys, there are 36 individually identifiable giant descending neurons. These identifiable descending neurons vary greatly in their regenerative abilities following a complete spinal cord injury, even when their axons run in similar paths in a spinal cord that is permissive for axonal regrowth. So, lampreys offer an interesting animal model to study the molecular mechanisms that underlie the ability of neurons to regenerate. The main disadvantage of the lamprey model is related to their life cycle. It takes between 5 and 7 years from birth to sexual maturity; therefore, it is impossible to generate stable mutant or transgenic lines in the laboratory. In any case, we have been able to implement both drug and genetic treatments (morpholinos) to be able to carry out these functional studies.

“Seeing a lamprey with a complete spinal cord injury that has completely recovered its swimming capacity never ceases to amaze me… The plasticity of the nervous system in these animals is amazing.”

**What has surprised you the most while conducting your research?**

Seeing a lamprey with a complete spinal cord injury that has completely recovered its swimming capacity never ceases to amaze me. Specifically, in this study, I was surprised to see that lampreys in which axon regeneration was inhibited with different treatments were still able to recover locomotion similarly to control animals. The plasticity of the nervous system in these animals is amazing.

**Figure:**
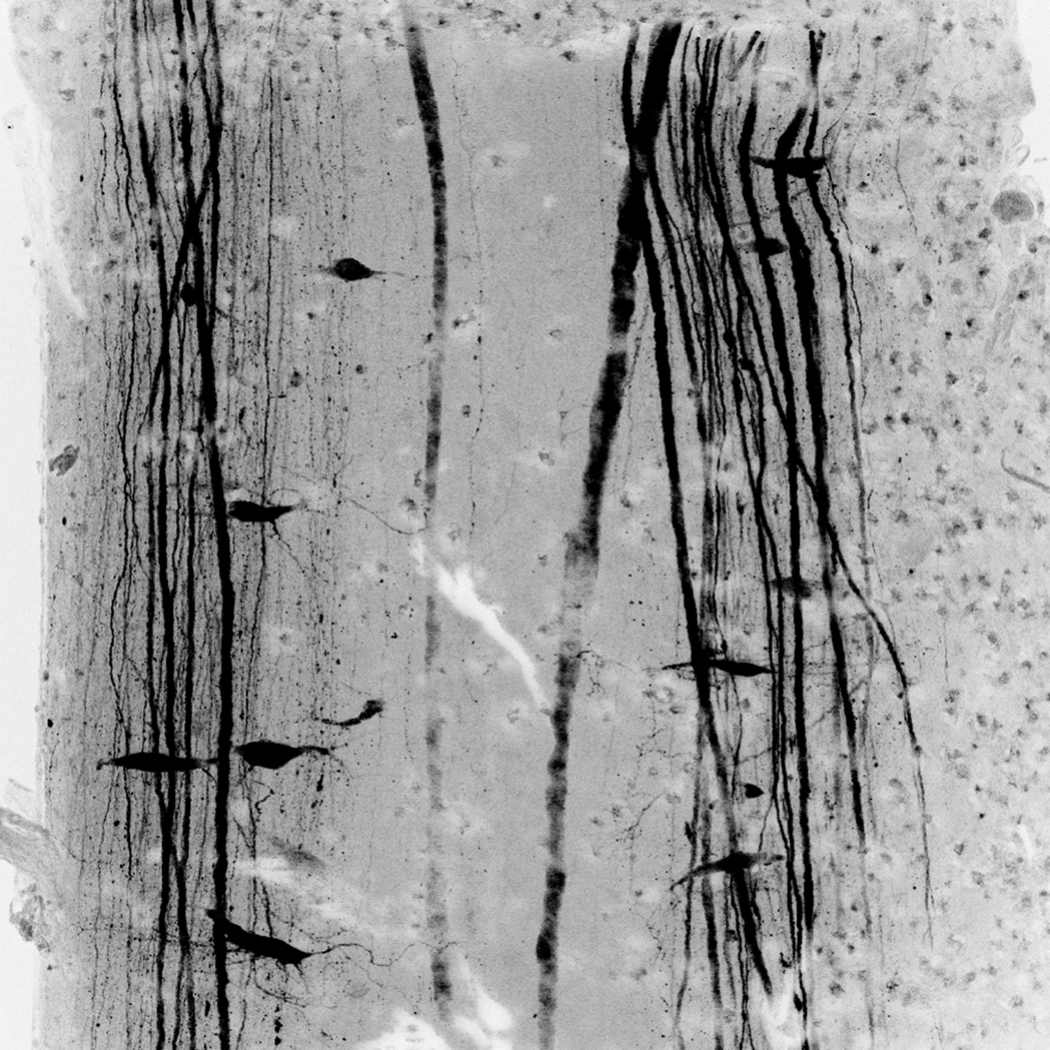
**Lamprey axons regenerated following a complete spinal cord injury.**

**Describe what you think is the most significant challenge impacting your research at this time and how will this be addressed over the next 10 years?**

There is no cure for spinal cord injury. Stem cells, tissue engineering, pharmacological treatments, physiotherapy, etc. have been investigated as a potential solution for spinal cord regeneration. However, I believe that there is strength in numbers and that combinatorial approaches are more likely to give the answer. The challenge is to combine the data provided by pharmacological studies, such as this, with advances in tissue engineering and other strategies. We must promote interdisciplinary research.

**What changes do you think could improve the professional lives of early-career scientists?**

The greatest difficulty in succeeding in a scientific career is the economic difficulty. Research is the basis of progress and development. But, many non-scientists are unaware of the work we do in the laboratory. I think that making society aware of research is key in leading countries to support young scientists.

“I think that making society aware of research is key in leading countries to support young scientists.”

**What's next for you?**

My next goal is to finish my PhD thesis in the following months. Then, I will look for an interesting place for a postdoctoral position that would allow me to continue studying the nervous system.

**Why did you choose Disease Models & Mechanisms to submit your paper?**

Lampreys are widely known as a model for evo-devo studies, but they are not so commonly used as a model for spinal cord regeneration. I like the focus of Disease Models & Mechanisms on the use of a wide variety of animal models, including non-conventional models like lampreys, to understand disease mechanisms and develop new treatments. I think that this journal brings together a very interesting niche where different models bring a variety ways of finding solutions to human diseases.
